# The introduction of LAG-3 checkpoint blockade in melanoma: immunotherapy landscape beyond PD-1 and CTLA-4 inhibition

**DOI:** 10.1177/17588359231186027

**Published:** 2023-07-17

**Authors:** Firas Y. Kreidieh, Hussein A. Tawbi

**Affiliations:** Department of Melanoma Medical Oncology, Division of Cancer Medicine, The University of Texas MD Anderson Cancer Center, Houston, TX, USA; Department of Melanoma Medical Oncology, Division of Cancer Medicine, The University of Texas MD Anderson Cancer Center, 1515 Holcombe Blvd, Houston, TX 77030, USA

**Keywords:** checkpoint inhibitor, ipilimumab, LAG-3, melanoma, nivolumab, relatlimab

## Abstract

Despite major advances with immunotherapy and targeted therapy in the past decade, metastatic melanoma continues to be a deadly disease for close to half of all patients. Over the past decade, advancement in immune profiling and a deeper understanding of the immune tumor microenvironment (TME) have enabled the development of novel approaches targeting and a multitude of targets being investigated for the immunotherapy of melanoma. However, to date, immune checkpoint blockade has remained the most successful with programmed cell death-1 (PD-1)/programmed cell death ligand-1 (PD-L1) and cytotoxic T-lymphocyte antigen-4 (CTLA-4) inhibitors, alone or in combination, yielding the most robust and durable clinical outcome in patients with metastatic melanoma. The highest rate of durable responses is achieved with the combination with PD-1 and CTLA-4 inhibition, and is effective in a variety of settings including brain metastases; however, it comes at the expense of a multitude of life-threatening toxicities occurring in up to 60% of patients. This has also established melanoma as the forefront of immuno-oncology (IO) drug development, and the search for novel checkpoints has been ongoing with multiple relevant targets including T-cell immunoglobulin and mucinodomain containing-3 (TIM-3), LAG-3, V-domain immunoglobulin suppressor T-cell activation (VISTA), T-cell immunoglobulin and immunoreceptor tyrosine-based inhibitory motif (ITIM) domain (TIGIT), among others. Lymphocyte activation gene-3 (LAG-3), which is a co-inhibitory receptor on T cells that suppress their activation, has revolutionized immunomodulation in melanoma. The ‘game changing’ results from the RELATIVITY-047 trial validated LAG-3 blockade as a relevant biological target and established it as the third clinically relevant immune checkpoint. Importantly, LAG-3 inhibition in combination with PD-1 inhibition offered impressive efficacy with modest increases in toxicity over single agent PD-1 inhibitor and has been U.S. Food and Drug Administration approved for the first-line therapy of patients with metastatic melanoma. The efficacy of this combination in patients with untreated brain or leptomeningeal metastases or with rare melanoma types, such as uveal melanoma, remains to be established. The challenge remains to elucidate specific mechanisms of response and resistance to LAG-3 blockade and to extend its benefits to other malignancies. Ongoing trials are studying the combination of LAG-3 antibodies with PD-1 inhibitors in multiple cancers and settings. The low toxicity of the combination may also allow for further layering of additional therapeutic approaches such as chemotherapy, oncolytic viruses, cellular therapies, and possibly novel cytokines, among others.

## Introduction

According to the Surveillance, Epidemiology, and End Results database, the 5-year relative survival rate for melanoma was 93.7% between 2012 and 2018 in the United States. Although it comprised only 1.3% of all cancer deaths, it is estimated that 99,780 new melanoma cases will be diagnosed in 2022, thus constituting 5.2% of all new cancer cases.^1^ In fact, according to the American Cancer Society, the melanoma age-standardized incidence rate has recently reached 22.7 per 100,000.^2^ With the rising incidence of melanoma, improvement of therapeutic options ensues.

Over the past decade, melanoma has been at the forefront of revolutionary advances in the field of immune checkpoint inhibitors (ICI). Long-term remissions provided by ICI even after stopping the medication suggested that they can have a curative potential for some patients with metastatic melanoma. Until 2010, the only therapies for melanoma approved by the U.S. Food and Drug Administration (FDA) have been chemotherapy with dacarbazine and high-dose IL-2. Approved in 1975 and 1998, respectively, dacarbazine and high-dose IL-2 remained the standard of care for metastatic melanoma based on their durable response rate, although this was only applicable to a small subset of patients. Neither of these agents, however, has shown an overall survival (OS) benefit in randomized trials.^3^

- While there are several potential targets under investigation, one of the most promising is LAG-3, which is a co-inhibitory receptor that suppresses T-cell activation and cytokine secretion.^4,5^ Approved in 2022 for the treatment of metastatic or unresectable melanoma in combination with nivolumab, relatlimab combined with nivolumab resulted in a superior progression-free survival (PFS) of 10.1 months, compared to 4.6 months for nivolumab monotherapy.^5^ In this review, we aim at providing an overview of LAG-3 immune checkpoint blockade, which extended the immunotherapy landscape in melanoma.

## CTLA-4 discovery initiates new era of checkpoint blockade

Seminal contributions to immune modulation using checkpoint blockade were made by Jim Allison and others, as they highlighted the role of T-cell priming and activation in mounting an immune response. These include the identification of receptor on T cells that recognizes and binds antigen and the discovery that T cells require a second molecular signal from the costimulatory molecule CD28 to launch a response to a bound antigen. In addition, the function of cytotoxic T-lymphocyte antigen-4 (CTLA-4) as a built-in off-switch on T cells was elucidated and CTLA-4 blocking antibodies were shown to unleash the T cells, thus enabling them to eliminate tumor cells.^6^ In March 2011, the anti-CTLA-4 antibody, ipilimumab, gained FDA approval for the treatment of metastatic melanoma based on evidence of improved OS in metastatic melanoma.^7^ In a randomized phase III trial that included patients with previously treated melanoma, ipilimumab, with and without a peptide vaccine, resulted in an improved OS with a median of 10.0 and 10.1 months, respectively, compared to 6.4 months in the vaccine-only group. Grade 3–5 immune-mediated adverse events occurred in 10–15% of patients. As such, despite the survival benefit, its use requires careful monitoring and may necessitate immune suppressive therapy.^8^

## PD-1 blockade improves clinical outcomes

In Keynote-001, 173 patients who progressed on ipilimumab were randomly assigned to receive either pembrolizumab 2 mg/kg every 3 weeks or 10 mg/kg every 3 weeks, with objective response rate (ORR) of 26% at both doses and with 58% and 63% of patients alive at 1 year, respectively. This was a remarkable finding for the anti-programmed cell death-1 (PD-1) antibody, pembrolizumab, particularly since the trial included heavily pre-treated individuals. Those findings resulted in the FDA approval of pembrolizumab.^9^ The efficacy and safety of another anti-PD-1 antibody, nivolumab, was studied in CheckMate-037, which included patients who were pre-treated with ipilimumab or with ipilimumab and v-raf murine sarcoma viral oncogene homolog B (BRAF) inhibitors. A total of 405 patients were randomized to nivolumab or chemotherapy with ORR of 31.7% and 10.6%, respectively. Grade 3 or 4 adverse events were reported in 9% of patients treated with nivolumab and 31% of those treated with chemotherapy.^10^

The CheckMate-067 trial by Wolchok *et al.* randomly assigned in 1:1:1 ratio patients with previously untreated advanced melanoma to receive nivolumab at a dose of 1 mg/kg plus ipilimumab at a dose of 3 mg/kg every 3 weeks for four doses, followed by nivolumab 3 mg/kg every 2 weeks, or nivolumab 3 mg/kg every 2 weeks plus placebo, or ipilimumab 3 mg/kg every 3 weeks for four doses plus placebo until progression. The median OS had not been reached in the nivolumab–ipilimumab combination group, was 37.6 months in the nivolumab group, and 19.9 months in the ipilimumab group. Moreover, the OS rate at 3 years was 58% in the nivolumab–ipilimumab combination group, 52% in the nivolumab group, and 34% in the ipilimumab group. However, ipilimumab–nivolumab combination has not shown a statistically significant improvement in OS over single-agent nivolumab.^11^ Grade 3 or 4 adverse events occurred in 59% of the patients in the combination group, as compared to 21% of those in the nivolumab group and 28% of those in the ipilimumab group.^12^ Single-agent immunotherapy, namely ipilimumab, pembrolizumab, or nivolumab, has shown efficacy for melanoma brain metastases (MBM), although less than extracranial effect.^13^ For example, patients with untreated MBM had a favorable response to ipilimumab with an ORR of 11% and up to 20% with nivolumab and pembrolizumab.^14,15^ To date, the most promising immunotherapy regimen for melanoma with brain metastases has been the combination of ipilimumab and nivolumab. The CheckMate-204 trial by Tawbi *et al.* reported 55% intracranial response rates, more than 85% of which were durable at 3 years, with equally impressive PFS, and OS for asymptomatic MBM patients further supported the first-line use of ipilimumab and nivolumab combination.

Despite the clinical success of ICI, however, melanoma tumor resistance remains a challenge leading to a decreased response rate.^16,17^ Moreover, while combination of approved ICI has shown remarkable efficacy, the cost of toxicity remained a challenge. Immune checkpoints that have been extensively studied in melanoma to date include PD-1/programmed cell death ligand-1 (PD-L1) and CTLA-4.^18^ To enhance the benefit from ICI in melanoma, there has recently been an evolving focus on identifying and targeting alternative novel immune checkpoints.^17^ LAG-3, which is a co-inhibitory receptor that suppresses T-cell activation and cytokine secretion, can be a promising immune checkpoint.^17,18^

## LAG-3 signaling

LAG-3 is a T-cell surface molecule that is closely related to cluster of differentiation CD4 with both genes located at the short arm of chromosome 12 and with proteins sharing 20% homology in amino acid sequence.^19^ Major histocompatibility complex (MHC) class I and class II proteins play an important role in the adaptive immune system. Although both classes share the task of presenting peptides on the cell surface for recognition by T cells, MHC I present peptides on nucleated cells and are recognized by cytotoxic CD8+ T cells, while MHC-II present peptides on antigen presenting cells (APC), including dendritic cells, macrophages, or B cells, and activate CD4+ T cells.^20^ Presentation of tumor antigens by APC through MHC-II to naïve T cells, by binding to T-cell receptor (TCR) and CD4, induces their activation. This MHC-T-cell signaling constitutes the initial step in T-cell activation. Immune checkpoints suppress T-cell activation in the tumor microenvironment (TME), which, in turn, results in suppressing clearance of tumor cells by the immune system.^17,18,21^

**Figure 1.**
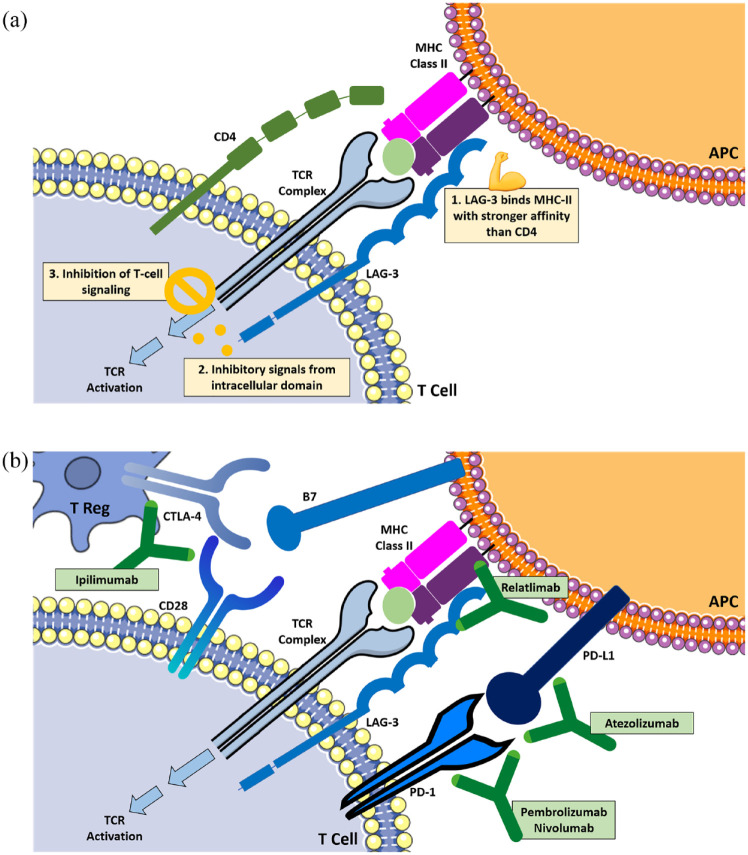
(a) Schematic overview of LAG-3 signaling pathway and (b) different checkpoints as immunotherapy targets.

Figure 1(a) depicts a schematic overview of LAG-3 signaling. Expressed on the cell membrane of tumor-infiltrating lymphocytes (TIL), on activated CD4+ and CD8+ T cells, and on regulatory T cells (Treg), LAG-3 binds to MHC-II on APC. This binding is similar to that of CD4 yet has a stronger affinity that is almost 100-fold.^18,19,22,23^ LAG-3 interacts with MHC-II and inhibits its binding to CD4 and TCR, thus inhibiting TCR signaling. Although LAG-3 recognizes and binds to MHC-II on APC just like CD4, the resulting blockage of T-cell activation is not only the result of competing with CD4, but also form inhibitory signals from its intracellular domains.^16^ Moreover, LAG-3 crosslinking with CD3 can impair T-cell proliferation and cytokine secretion by inhibition of influx of calcium. While its exact signaling mechanism has not yet been fully unraveled, the unique cytoplasmic tail of LAG-3 that is different from other immune checkpoints suggests unique molecular characteristics and role as compared to other immune checkpoints.^23,24^

Aberrant expression of LAG-3 was identified in a variety of tumors, including melanoma, and was associated with gain of function, evasion of tumor cells from the immune control system, and more aggressive disease.^21,24–27^ Also, overexpression of LAG-3 in T cells can provide protection to melanoma cells and can prevent apoptosis of tumor cells. Interestingly, there is increasing evidence that LAG-3 and the inhibitory immune checkpoint PD-1/PD-L1 can be extensively co-expressed on TIL, CD4+, and CD8+ T cells with striking synergy. In fact, dual genetic knockout of both LAG-3 and PD-1, in murine melanoma models, resulted in delayed growth of the tumor and increased survival of mice. This preclinical data offered potential therapeutic options by dual targets when blocking immune checkpoints in melanoma, thus overcoming resistance to single-agent treatment.^17,28^

In addition, LAG-3 can also intersect with the CTLA-4 signaling pathway. Both LAG-3 and CTLA-4 can inhibit TCR signaling pathway, thus resulting in immune tolerance of tumor cells. For example, in mice models with anterior chamber-associated immune deviation, which is a model of systemic immune tolerance characterized by an antigen-specific suppression of delayed type hypersensitivity, the frequency of both LAG-3 and CTLA-4 on Treg was high.^29^ Interestingly, a recent study investigating the effect of ipilimumab, which is a CTLA-4 antibody, showed that ipilimumab can result in an increased expression of LAG-3 in TIL in metastatic melanoma patients, thus suggesting intersection of both pathways.^16^ Currently, there is a variety of ongoing clinical trials that explore this therapeutic backbone of combination immunotherapy simultaneously targeting LAG-3 and PD-1, or CTLA-4.^17,28,30^Figure 1(b) depicts a schematic overview of different checkpoints as immunotherapy targets.

## The emerging role and clinical outcomes of LAG-3 inhibition

LAG-3 inhibition is an emerging promising immune checkpoint target in melanoma. Antibodies targeting LAG-3 can result in promoting effector TCR signaling pathway and inhibit suppression by Treg cells. Moreover, the possible interaction between LAG-3 and other checkpoints, namely PD-1 and CTLA-4, holds significant promise in raising the bar and extending the landscape of melanoma immunotherapy.^17,31,32^

Antagonist anti-LAG-3 antibodies have been the mainstay of studies interested in releasing the brakes of the immune system in melanoma. These antibodies include BM-986016, REGN3767, and TSR033.^33^ The first anti-LAG-3 antibody relatlimab, BMS-986016, was developed in 2013 and is currently undergoing evaluation in more than 12 phase I and II clinical trials in hematological and solid tumors.^33,34^ The first report on efficacy of relatlimab in melanoma was introduced through a phase I/II clinical trial (NCT01968609). Ascierto *et al.* evaluated the combination of relatlimab and nivolumab, an anti-PD-1 agent, in 68 patients with melanoma whose disease was refractory to prior anti-PD-1/PD-L1 therapy. This dose-escalation trial showed that patients with LAG-3-expressing tumors had a greater overall response rate with a well-tolerated safety profile that was similar to nivolumab monotherapy.^35^ Updated data from the 2017 European Society of Medical Oncology (ESMO) Congress showed a further increased ORR reaching 18% in patients with LAG-3-positive tumors. Enhanced response was associated with greater LAG-3 expression and independent of PD-L1 expression. LAG-3 expression more than or equal to 1% was more likely to benefit from the combination therapy of relatlimab and nivolumab.^35,36^

The RELATIVITY-047 trial evaluated the same combination of relatlimab and nivolumab in comparison *versus* single-agent nivolumab in patients with metastatic melanoma who were treatment naïve. Interim results were presented at the 2021 annual meeting of the American Society of Clinical Oncology (ASCO) and published by Tawbi *et al.* in 2022.^37^ This phase III trial included 714 patients with previously untreated metastatic melanoma and who were treatment naïve. Patients were randomized to receive either single-agent nivolumab at a dose of 480 mg or a combination of nivolumab (480 mg) and relatlimab (160 mg) every 4 weeks. The primary end point was PFS, and results presented at median follow-up of 13.2 months showed a median PFS of 10.1 months, which was significantly greater than that of single-agent nivolumab group, 4.6 months (*p* = 0.0055). PFS at 12 months was 47.7% with the combination arm compared to 36.0% for the nivolumab arm.^5,37^

The improved PFS was independent of the LAG-3 or PD-L1 expression status and was present across all prespecified subgroups.^5,37^ It is worth noting that patients who had baseline characteristics that are usually associated with worse prognosis, including visceral metastases, high tumor mutation burden, increased serum lactate dehydrogenase, having mucosal melanoma, showed a superior outcome with combination therapy when compared to single-agent nivolumab. Moreover, benefit of the combination therapy was observed in both, patients who had BRAF mutation and those who were BRAF wild type.^5^ Expression of LAG-3 ⩾ 1% and PD-L1 ⩾ 1% was detected in the TME of 75.2% and 41.0% of the patients. Among the 25% of patients with less than 1% LAG-3-positive cells, the hazard ratio (HR) was 0.78, which was still in favor of the combination of relatlimab and nivolumab, yet with a wide confidence interval (0.54–1.15), which was most likely related to the small number of patients in that subgroup.^5,37^

Further analyses presented at the ASCO March 2022 plenary series with median follow up of 19.3 months showed a slight shift in median PFS to 10.2 *versus* 4.6 months for patients who received nivolumab, and ESMO 2022. Updated results included secondary end points, such as OS and overall response rate. The OS was not yet reached with relatlimab and nivolumab but was 34.1 months with nivolumab alone. The difference between the two arms missed the significance threshold, which was defined as two-sided *p* < 0.04302 based on 69% power of target HR of 0.75.^38^ Despite this lack of significance in OS, as additional events occur in the future years, significance may be seen. ORR was 43.1% for those who received relatlimab and nivolumab compared to 32.6% for those who received nivolumab alone. The combination group was associated with grade 3 or 4 adverse events in 18.9% of patients, compared to 9.7% for the nivolumab group. Although more toxicity was seen in the combination group, the safety profile was still considered manageable with no new unexpected safety alarm signals.^5,38^ Additional studies are needed to understand the efficacy of relatlimab–nivolumab in patient populations that are usually excluded from clinical trials for the treatment of melanoma, such as patients with active or untreated brain metastasis or with rare subtypes of melanoma, including uveal melanoma.^37^

Targeting two checkpoints in melanoma has been a well-established treatment option with good long-term OS. The CheckMate-067 trial showed a durable response and significantly improved OS with dual checkpoint inhibition with CTLA-4 inhibitor and PD-1 inhibitor in patients with treatment-naïve metastatic melanoma.^12,39^ This trial led to the FDA approval of the combination therapy of ipilimumab and nivolumab, which became the standard of care for metastatic melanoma in the first-line setting.^12,39–42^ RELATIVITY-047 trial compared dual checkpoint inhibition with relatlimab and nivolumab to nivolumab alone, similar to the comparison made in the CheckMate-067 trial between the two arms. This allowed for direct comparison of dual checkpoint inhibition to single-agent immunotherapy in metastatic melanoma in both trials. Moreover, the PFS benefit observed in the combination group of relatlimab and nivolumab in RELATIVITY-047 trial (47.7%) was comparable to that observed in the combination group of ipilimumab and nivolumab group in CheckMate-067 trial (49%). However, this similarity should be interpreted with caution as cross-trial comparison is not allowed.^12,39–41^

The results from RELATIVITY-047 trial were further reinforced by a trial that investigated the combination of relatlimab and nivolumab, yet in the neoadjuvant setting. This was important as it allowed the evaluation of treatment efficacy rapidly, post-surgical resection at 6–8 weeks following initiation of therapy.^43^ In all, 29 patients with stage IIIB–IV resectable melanoma were included in this trial. Pathological response at the time of surgical resection, 8 weeks after treatment initiation, was evaluated. Treatment consisted of two sessions of relatlimab and nivolumab at the same doses of the RELATIVITY-047 trial. Following surgical resection, patients received the same combination regimen every 4 weeks for 10 months as an adjuvant therapy. While the study included only 29 patients, complete response (59%) and nearly complete response (7%) were very exciting results. 26% of patients experienced grade 3 or 4 adverse events, all of which were manageable and tolerable. At median follow-up of 16.2 months, all 19 patients who had complete response or nearly complete pathological response did not have disease relapse.^43^

While relatlimab and nivolumab are given continuously and as a single infusion, the standard of care ipilimumab and nivolumab combination consists of induction therapy with both agents followed by nivolumab alone and stopping ipilimumab.^5,41^ More studies are needed to determine whether relatlimab and nivolumab can be although this may not be a direct extrapolation due to the potentially different mechanisms of response and resistance to relatlimab and ipilimumab and their distinct modes of action.^5,16,38,41^ There are several ongoing clinical trials that investigate the novel combination of nivolumab–relatlimab in various clinical settings, including both first-line and PD-1 refractory settings. Both immune checkpoint combinations, ipilimumab–nivolumab and nivolumab–relatlimab, have been investigated in previously untreated patients. Consider the difference in their toxicity profile, head-to-head comparison through a clinical trial is unlikely. With few data on the activity of nivolumab–relatlimab after progression in patients previously treated with ipilimumab–nivolumab, and vice versa, the decision regarding first-line treatment and the appropriate sequence for each patient needs to be defined.^44^

A small retrospective study demonstrated low efficacy of ipilimumab–nivolumab combination following progression in patients who received nivolumab–relatlimab in the first-line setting.^45^ In patients with PD-1 refractory disease, nivolumab–relatlimab combination is associated with lesser efficacy and an overall response rate of 11.5%.^35^ Of the 43 enrolled patients, 30 (70%) had prior anti-CTLA-4 treatment, and patients with LAG-3 expression in the TME that was at least 1% showed significantly higher responses than patients with LAG-3 expression less than 1% (ORR 18% *versus* 5%). As for the first-line setting, while there was a treatment benefit for the combination over nivolumab monotherapy, regardless of LAG-3 expression, patients who had LAG-3 that was at least 1% had longer PFS with mean PFS of 12.58 months.^5^ As such, LAG-3 expression may only be an ancillary biomarker that is not essential when making treatment decision. The efficacy of nivolumab–relatlimab combination for previously treated patients is being studied in several clinical trials, namely NCT01968109 and NCT03484923, and in triple combination with anti-CTLA-4 in NCT03459222.^44^

## Other approaches to targeting the LAG-3 pathway

Investigating the role of LAG-3 as an immuno-modularity target began in 2006 with IMP321, which is the LAG-3Ig fusion protein, a soluble dimeric fusion protein consisting of four LAG-3 extracellular domains.^16,46^ After it was used initially in mice to induce antitumor response, several studies were conducted about its role for renal cell carcinoma, metastatic breast cancer, and melanoma.^46–48^ Romano *et al.* included 12 patients with advanced melanoma who received a lymphodepleting non-myeloablative conditioning chemotherapy and autologous peripheral blood mononuclear cells, followed by vaccination with MART-1 peptide vaccination. They investigated the role of adding adjuvant IMP321 to the melanoma-associated antigen recognized by T-cells (MART-1) peptide vaccine with a hypothesis that this combination would induce a long-lasting antitumor immunity and improve patient outcome. Interestingly, the addition of IMP321 resulted in a durable antitumor immune response with a significant reduction in Treg cells production.^48^ The future implications of IMP321 are further discussed in a following section.

In a phase I/IIa clinical trial by Legat *et al.*,^16^ metastatic melanoma patients received vaccines that included IMP321, Montanide ISA-51, and five synthetic peptides. IMP321 acted as an APC activator and decreased Treg immunosuppressive effect, thus allowing optimal antigen presentation to CD8+ T cells. IMP321 induced specific CD4+ T cells response in all 16 patients and specific CD8+ T cells response in 13 patients with a favorable safety profile.^22^ A currently phase I dose-escalation clinical trial evaluated the role of LAG-3 fusion protein, combined with pembrolizumab, in metastatic melanoma. Half of the 18 patients had significant tumor reduction, and the combination had an acceptable safety profile.^49,50^

## Potential novel ICI in melanoma

Advancement in immune profiling and a deeper understanding of the immune TME have enabled the development of novel approaches to enhance the antitumor immune response. This has allowed attempts to utilize the interaction between its various components, including TIL and the extracellular matrix, to develop better therapeutic options. Recent data have identified a multitude of agonist and inhibitory receptors that have been pursued as therapeutic targets for advanced melanoma. Costimulatory receptors of the tumor necrosis factor (TNF) receptor superfamily, including OX-40, CD27, and glucocorticoid-induced TNF receptor, have been targeted with the use of agonistic antibodies to promote antitumor T-cell responses. They have shown weak or non-durable responses, however, according to results from phase I clinical trials whereby they were used as monotherapy.^51–54^ On the other hand, inhibitory immune checkpoints, namely T-cell immunoglobulin and mucinodomain containing-3 (TIM-3), T-cell immunoglobulin and ITIM domain (TIGIT), and V-domain immunoglobulin suppressor T-cell activation (VISTA), have also been targets of interest in melanoma.^55^

TIM-3, which is also referred to as hepatitis A virus cellular receptor 2, is a type I transmembrane protein whose extracellular domain consists of the N-terminal immunoglobulin (IgV) domain located at the distal end of the membrane followed by the membrane mucin domain that contains an O-linked glycosylation potential. Expressed on CD4+ and CD8+ T cells, Treg, natural killer cells, dendritic cells, and Th17 cells, TIM-3 binds to a wider spectrum of ligands on normal and malignant cells, including galectin-9 and phosphatidylserine.^56–58^ Similar to LAG-3, TIM-3 is also involved in exhaustion of T cells, which results in failure of T cells to proliferate and exert their effector function, including cytokine release and cytotoxicity.^59^

Co-expression of TIM-3 and PD-1 on TIL in mice with solid tumors, including B16F10 melanoma, was reported by Sakuishi *et al.* They showed that the most abundant cell population were CD8+ TIL co-expressing TIM-3 and PD-1. Interestingly, while treatment with antibodies targeting TIM-3 alone did not affect tumor growth and treatment with anti-PD-L1 antibodies showed delayed tumor growth, treatment with antibodies targeting both resulted in a significant tumor growth reduction whereby almost half of mice showed complete regression of their tumors.^60^ This suggested that increased expression of TIM-3 in TIL might have a promising predictive and prognostic value.

To date, there are several clinical trials that combine ICI and that include antibodies against TIM-3 in solid tumors. Curigliano *et al.* study sabatolimab, an anti-TIM-3 antibody, with or without spartalizumab, an anti-PD-1 antibody. While patients receiving sabatolimab had no response, five patients receiving combination therapy had partial response one of whom had malignant perianal melanoma. The authors suggested that combining sabatolimab and spartalizumab results in enhanced antitumor activity.^61^ This combination was studied in another phase II trial, NCT02608268, which included 16 patients with melanoma.^62^ In addition, a phase I/II trial NCT04370704 is studying the combination of antibodies against PD-1 (INCMGA00012), LAG-3 (INCAGN02385), and TIM-3 (INCAGN02390) in selected tumors, including melanoma.^63^

In addition to anti-TIM-3 antibodies, TIGIT, which is mainly expressed on regulatory and memory T cells and natural killer cells, was also introduced in 2009 as T-cell activation suppressor.^64^ TIGIT binds two main ligands, namely CD155 and CD112, and competes with other counterparts, namely CD266 and CD96, thus exerting an immunosuppressive effect on T cells. While CD266 delivers a positive costimulatory signaling pathway, TIGIT delivers inhibitory signals.^65^ In human and mice models, ligation of TIGIT can result in inhibition of natural killer cells cytotoxicity through its ITIM cytoplasmic domain.^65^ Moreover, preclinical studies showed synergistic effects between TIGIT and anti-PD-1 antibodies, which can, in turn, increase the antitumor effect of CD8+ T cells.^66^ The first human study that targets TIGIT was conducted by Niu *et al.* and showed that vibostolimab, an antibody against TIGIT, exhibited an improved antitumor activity when combined with an anti-PD-1 antibody, pembrolizumab, with an acceptable toxicity profile in solid tumors.^67^ In melanoma, the development of anti-TIGIT and its combination with other ICI, including LAG-3, are still underway. To our knowledge, there is one phase I/II trial that is investigating the role of combining vibostolimab or lenvatinib, an anti-VEGF antibody, with pembrolizumab and quavonlimab, an anti-CTLA-4 antibody, in patients with PD-1 refractory disease (NCT04305041).^44^

Recent clinical trials have also investigated the use of combination antibody therapies that target VISTA. Anti-VISTA antibodies appear to target a pathway that does not overlap with the CTLA-4 and PD-1/PD-L1 pathways,^68,69^ and some studies have shown that negative immune checkpoint regulation by VISTA represents an important potential mechanism of acquired resistance in melanoma patients who are pre-treated with anti-PD-1.^70,71^

## Future implications for clinical practice

Dual checkpoint inhibition with CTLA-4 and PD-1 inhibitors has become a well-established therapeutic option for metastatic melanoma with long-term outcomes, yet at the expense of toxicity with more than half of patients receiving ipilimumab and nivolumab having grade 3 or 4 adverse events. The results from the RELATIVITY-047 trial represent a new breakthrough in the era of checkpoint inhibition and are definitely practice changing. They validated the option of LAG-3 blockade, in combination with PD-1 inhibitor, as a therapeutic option for patients with melanoma. This also introduced LAG-3 as the third immune checkpoint in the landscape of melanoma immunotherapy. In fact, on March 18, 2022, the FDA-approved fixed doses combination of relatlimab and nivolumab for adults and pediatric patients with unresectable or metastatic melanoma.^72,73^

Data obtained from RELATIVITY-047 lack the sufficient maturity for providing a final interpretation regarding the OS benefit of this new combination. In addition, the clinical conundrum will remain as to which combination to choose for an individual patient, and until prospective randomized studies are conducted to compare the two checkpoint inhibitor combination this will remain a highly personalized decision based on clinical characteristics of the patient.^5,37,38^ Naturally, as this combination was recently approved, more extensive data exist for ipilimumab and nivolumab combination as compared to relatlimab and nivolumab combination.^5,16,74^ In addition, determining the population of melanoma patients who would benefit the most from relatlimab combination therapy remains an important subject for future studies. The different modes of actions also raise the question of whether triple therapy is a possibility with relatlimab, ipilimumab, and nivolumab, but this has to be considered with caution as it can carry risk for greater toxicity.

On the other hand, more studies are needed to investigate the role of relatlimab in earlier stages of the disease including the adjuvant and neoadjuvant settings for patients with stage III disease. The neoadjuvant setting is particularly important in melanoma as the TME is usually intact as compared to advanced disease and can, therefore, enable scientists to better identify patients with pathologic complete response rate and to determine factors that can be associated with better clinical outcomes and better understand the mechanisms of response to treatment, resistance, and micrometastases by analyzing collected surgical specimens.^16,74^

LAG-3 inhibitors can directly bind LAG-3 molecules or their ligands, thus blocking their interaction between ligands and LAG-3 and downregulating the inhibitory efficacy of LAG-3 toward the immune system. Not only anti-LAG-3 antibodies restore T-cell function, but also they inhibit Treg activity. Studies have shown that antibodies against PD-1 can only activate T cells but cannot inhibit Treg. As such, LAG-3 inhibitors remain a promising novel tumor immunotherapy target beyond PD-1/PD-L1 and CTLA-4 inhibition.^75–78^ The findings of addition of relatlimab to the immunotherapy backbone in melanoma have accelerated other research studies to investigate further LAG-3-directed therapy combinations. Nagasaki *et al.* demonstrated a far greater efficacy for combination treatment with anti-PD-1 and anti-LAG-3 antibodies on MHC-II expressing tumors than either agent alone. LAG-3 inhibits the antitumoral effect of anti-PD-1 and anti-LAG-3 therapy in Hodgkin lymphoma by inhibiting the CD4+ T-cell response. Bispecific antibodies have become a new subject of future research due to flexible pathways of functioning. With Fc-mediated immune activity, bispecific antibodies exhibit greater potential for antitumor immunotherapy.^79,80^ For example, the phase II PLATforM trial (NCT03484923) evaluated LaG-525, a monoclonal antibody against LAG-3, in combination with spartalizumab, a monoclonal antibody against PD-1.^81,82^ In fact, there are around 50 currently ongoing clinical trials that evaluate the efficacy of adding antibodies against LAG-3 to other drugs.^83^ These include a phase I trial that assesses a bispecific antibody targeting both, LAG-3 and PD-1 (RO7247669) in solid tumors that are refractory to prior therapy (NCT04140500).^84^ Other bispecific antibodies against LAG-3 and PD-1 include MGD013 and FS118, which are currently ongoing investigation in phase I clinical trials.^17^

In addition to LAG-3 antagonists, the use of IMP321 in combination with pembrolizumab, an anti-PD-1, is also under investigation in advanced melanoma (NCT02676869).^85^ IMP321, also known as Eftilagimod alpha, is a soluble version of anti-LAG-3. Interestingly, it targets antigen-presenting cells and transduces an MHC-II-mediated feedback signal. This results in increased T-cell proliferation and a full cytotoxic T cells activated phenotype characterized by increased production of IFN-γ, TNF-α, and IL-6. It also promotes production of CCL4 and TNF-α by myeloid cells. Soluble LAG-3 fusion protein enhances the capacity of MHC-II macrophages or immature dendritic cells to induce T-cell responses whereby tumor regression involves the recruitment of CD8+ T-cell response. There remain several unanswered questions regarding anti-LAG-3 treatment approach, including the impact of anti-LAG-3 and anti-PD-1 therapy in other settings, such as brain metastasis, adjuvant stage II/III melanoma, and rare melanoma subtypes, namely acral, mucosal, uveal, and desmoplastic melanoma. So much so, a new phase II clinical trial that studies nivolumab–relatlimab combination in patients with active MBM was recently initiated by Tawbi *et al.* at the University of Texas MD Anderson Cancer Center.^86^ To date, there are 13 ongoing clinical trials involving LAG-3-IG fusion protein. Its safety and tolerability combined with its efficacy support the future development of this drug for clinical use in combination with first-line regimens.^48,80,87,88^ As the first commercially developed anti-LAG-3 antibody, relatlimab was first studied in clinical trials in 2013.^89^ Due to its limited efficacy as single agent, relatlimab is generally being studied in combination with other checkpoint inhibitors, including CTLA-4 and PD-1 inhibitors, and currently has 46 different clinical trials for cancer therapy.^87,90^Tables 1 and 2 show the currently active ongoing clinical trials that investigate LAG-3-based therapies for non-melanoma and melanoma tumors.^36^

**Table 1.** Active ongoing LAG-3-based clinical trials for non-melanoma solid tumors.

LAG-3-based therapy	Clinical trial	NCT number	Tumor	Recruiting?
Nivolumab/relatlimab	Phase I	NCT04658147	Potentially resectable hepatocellular carcinoma	Yes
	Phase I	NCT02966548	Advanced solid tumors	No
	Phase I	NCT03044613	Potentially resectable gastric cancer	No
	Phase I/II	NCT03459222	Advanced solid tumors	Yes
	Phase I/II	NCT02488759	Advanced solid tumors	No
	Phase I/II	NCT03610711	Advanced gastroesophageal cancer	Yes
	Phase I/II	NCT04611126	Advanced ovarian cancer	Yes
	Phase I/II	NCT05134948	Advanced solid tumors	Yes
	Phase I/II	NCT05337137	Advanced hepatocellular carcinoma	Yes
	Phase II	NCT04095208	Advanced soft tissue sarcoma	Yes
	Phase II	NCT03623854	Advanced chordoma	Yes
	Phase II	NCT04080804	Head and neck squamous cell carcinoma	Yes
	Phase II	NCT03607890	Advanced mismatch repair deficient solid tumors	Yes
	Phase II	NCT03642067	Microsatellite Stable colorectal adenocarcinoma	Yes
	Phase II	NCT04567615	Hepatocellular carcinoma	Yes
	Phase II	NCT03521830	Advanced basal cell carcinoma	Yes
	Phase II	NCT04326257	Head and neck squamous cell carcinoma	Yes
	Phase II	NCT04623775	Non-small-cell lung cancer	Yes
	Phase II	NCT04205552	Non-small-cell lung cancer stage I/II/IIIA	Yes
	Phase II	NCT03867799	Metastatic colorectal cancer	No
	Phase II	NCT05148546	Resectable clear cell renal cell carcinoma	Yes
	Phase II	NCT04062656	Advanced gastric cancer	Yes
	Phase III	NCT05328908	Advanced colorectal cancer	Yes
Tebotelimab (PD-1 × LAG-3 bispecific molecule)	Phase I	NCT03219268	Her2+ advanced solid tumors	No
	Phase II	NCT04634825	Head and neck squamous cell carcinoma	Yes
	Phase II/III	NCT04082364	Her2+ advanced gastric cancer	No
RO-7247669 (PD-1 × LAG-3 bispecific molecule)	Phase I	NCT04140500	Advanced solid tumors	Yes
	Phase I/II	NCT04524871	Advanced liver cancers	Yes
	Phase II	NCT04785820	Advanced esophageal squamous cell carcinoma	Yes
Favezelimab (anti-LAG-3 antibody)	Phase I/II	NCT04938817	Advanced small-cell lung cancer	Yes
	Phase I/II	NCT04626479	Advanced clear cell renal cell carcinoma	Yes
	Phase I/II	NCT04626518	Advanced clear cell renal cell carcinoma	Yes
	Phase II	NCT04895722	Mismatch repair deficient/MSI high advanced colorectal cancer	Yes
	Phase II	NCT03516981	Advanced non-small-cell lung cancer	No
	Phase III	NCT05064059	Advanced colorectal cancer	Yes
	Phase III	NCT05064059	Advanced colorectal cancer	Yes
EMB-02 (PD-1 × LAG-3 bispecific molecule)	Phase I/II	NCT04618393	Advanced solid tumors	Yes
FS 118 (PD-L1 × LAG-3 bispecific molecule)	Phase I/II	NCT03440437	Advanced solid tumors	Yes
IBI-323 (PD-L1 × LAG-3 bispecific molecule)	Phase I	NCT04916119	Advanced solid tumors	Yes
HLX 26 (anti-LAG-3 monoclonal antibody)	Phase I	NCT05078593	Advanced solid tumors	Yes
LBL-007 (anti-LAG-3 monoclonal antibody)	Phase I/II	NCT05102006	Advanced solid tumors	Yes

PD-1, programmed cell death-1; MSI, microsatellite instability.

**Table 2.** Active ongoing LAG-3-based clinical trials for melanoma.

LAG-3-based therapy	Clinical trial	NCT number	Melanoma	Recruiting?
Nivolumab/Relatlimab	Phase I/II	NCT03978611	Advanced melanoma	Yes
	Phase II	NCT04552223	Metastatic uveal melanoma	Ye
	Phase II	NCT03743766	Advanced melanoma	Yes
	Phase II	NCT05002569	Resected stage III–IV melanoma	Yes
	Phase II	NCT02519322	Stage IIIB–IV melanoma	No
	Phase II/III	NCT03470922	Advanced melanoma	No
RO-7247669 (PD-1 × LAG-3 bispecific molecule)	Phase I/II	NCT05116202	Advanced melanoma	Yes
LBL-007 (anti-LAG-3 monoclonal antibody)	Phase I	NCT04640545	Advanced melanoma	Yes

PD-1, programmed cell death-1.

## Conclusions

Melanoma has been at the forefront of immunotherapy with at least three checkpoint targets to date, namely PD-1, CTLA-4, and LAG-3, being first FDA approved in metastatic disease. While dual checkpoint inhibition with CTLA-4 and PD-1 inhibitors has become a well-established therapeutic option for metastatic melanoma with long-term OS results, this came at the expense of toxicity. The ‘game changing’ results from the RELATIVITY-047 trial were revolutionary and validated the option of LAG-3 blockade, in combination with PD-1 inhibitor, as a therapeutic option for patients with melanoma.^28–30^

While awaiting results from ongoing trials, data available from the RELATIVITY-047 trial have shown significant improvement in PFS with combining relatlimab and nivolumab in metastatic melanoma with a tolerable safety profile. The challenge remains to elucidate the efficacy of this combination in patients with untreated brain or leptomeningeal metastases or with rare melanoma types, such as uveal melanoma. The findings of addition of relatlimab to the immunotherapy backbone in melanoma shall accelerate other research studies to investigate further these patient populations and to better understand LAG-3-directed therapy combinations. Also, better insights into the impact of LAG-3 inhibition on effector T cells and other immune cell populations in the TME shall be a major priority across the melanoma immuno-oncology discipline and can help identify predictive biomarkers to evaluate response to treatment and identify patients who would most likely benefit from this combination therapy.

## Declarations

**Ethics approval and consent to participate:** Not applicable.

**Consent for publication:** Not applicable.

**Availability of data and materials:** Available upon request.
